# Unravelling the difference in craniofacial morphology of Yucatan miniature and standard pigs during postnatal ontogeny

**DOI:** 10.1098/rspb.2025.1646

**Published:** 2025-08-20

**Authors:** Ce Liang, Tengyang Qiu, Arsalan Marghoub, Damith S. Chathuranga, Costantino Buzi, Antonio Profico, Marius Didziokas, Michael C. Baldwin, Erwin Pauws, Zi-Jun Liu, Katherine L. Rafferty, Susan W. Herring, Mehran Moazen

**Affiliations:** ^1^ Department of Mechanical Engineering, University College London, London WC1E 7JE, UK; ^2^ Developmental Biology and Cancer Research & Teaching Department, Great Ormond Street Institute of Child Health, University College London, London WC1N 1EH, UK; ^3^ DFG Centre of Advanced Studies ‘Words, Bones, Genes, Tools’, University of Tübingen, Tübingen 72074, Germany; ^4^ Department of Physics and Geology, University of Perugia, Perugia 06123, Italy; ^5^ Department of Biology, University of Pisa, Pisa 56126, Italy; ^6^ Department of Oral Health Sciences, School of Dentistry, University of Washington, Seattle 98195-7446, USA; ^7^ Department of Orthodontics, School of Dentistry, University of Washington, Seattle 98195-7446, USA

**Keywords:** domestic pig, miniature swine, suture maturation, calvarial thickness, cranial base, growth trajectory

## Abstract

The Yucatan miniature pig has become a preferred model for craniofacial research due to its anatomical and physiological similarities to humans. However, the factors driving midfacial hypoplasia in Yucatans during postnatal ontogeny remain unclear. This study characterized postnatal skull growth and development, and morphological variations in Yucatan and standard (domestic) pigs, with a focus on the role of joint maturation in resulting craniofacial dysmorphology. Forty available head specimens (Yucatan: *n* = 20, 0–12 months; standard: *n* = 20, 0–8 months) were analysed using multidimensional craniometric measurements and geometric morphometrics. Results show that both breeds follow an allometric growth trajectory, largely driven by the expansion of craniofacial organs and capsules. At birth, skull morphology was similar between breeds; however, Yucatans developed a shorter face and more compact neurocranium, while maintaining a nearly identical mandibular shape to standard pigs. Over the first three months, Yucatans exhibit delayed calvarial suture fusion compared with standard pigs, while skull base synchondroses remain patent in both breeds. These findings reflect the scaling relations between breeds and highlight differential growth patterns of the midface, neurocranium and mandible in Yucatans, emphasizing their interactions with organ development, cavity expansion and joint maturation, offering insights into the mechanisms driving craniofacial diversification in pig models.

## 1. Introduction

Pigs now serve as an essential animal model in studying the developmental processes, underlying mechanisms of congenital diseases, and musculoskeletal biomechanics, as well as developing effective therapeutics and vaccines, owing to their anatomical and physiological similarities to humans [1–5]. Pig models, especially miniature pigs, show substantial potential in the field of craniofacial research, considering their comparable craniofacial size, dentition and masticatory mechanics [6–8]. While rodent models are widely used in investigating the genetic mechanisms of craniofacial development and disease, their craniofacial structure and growth patterns differ from humans [3,9]. Hence, these attributes make pig models superior to rodent models for comparative studies in craniofacial biology.

In recent years, the Yucatan miniature pig has become a preferred animal model for laboratory use [4,10]. Compared with large pig breeds (i.e. Yorkshire, Landrace, Duroc and wild boar), Yucatans have been selected for small body size with a genetically controlled growth rate [11]. Sexual maturity in Yucatan pigs occurs around 4.4 [12] to 6 months of age (reported by Premier Biosource Inc.), similar to standard (domestic) pigs, which typically reach sexual maturity between 4.5 and 6 months [4]. Artificial selection in Yucatans has led to a midfacial hypoplastic head along with a more compact body structure compared with standard pigs. However, the mechanisms of these phenotypic changes in the craniofacial dysmorphology of Yucatan pigs during postnatal ontogeny remain unclear.

Experimental data suggest that the midfacial hypoplasia in Yucatans likely arises from a complex interplay of genetic, developmental and biomechanical factors [11,13–15]. However, the question of which factor plays a primary role in altering the growth dynamics of the craniofacial skeleton during postnatal ontogeny is still open. One major limitation in addressing this question is the scarcity of studies that systematically quantify morphological changes in pig skulls across different breeds and developmental stages. Most studies have focused on a single breed using traditional craniometric measurements [16,17], with less effort to investigate the complex three-dimesional craniofacial growth patterns between breeds (e.g. [18,19]). Furthermore, as inspired by previous research on humans [20] and mice [21], it would be of great interest to evaluate how the pig craniofacial system adapts to the differential growth of key anatomical structures, such as brain, eye and nasal cavity, and to assess the contributions of early ossification patterns, particularly in calvarial and skull base joints. Expanding such analyses across multiple pig breeds, including Yucatan and standard pigs, will not only improve our understanding of breed-specific craniofacial development in pigs but also offer insights into the mechanisms of driving postnatal craniofacial dysmorphology in humans (e.g. craniosynostosis), as well as in other suids and mammals with shared developmental traits.

The overall aim of this study was to carry out a detailed characterization of postnatal craniofacial system growth and development in Yucatan and standard pigs, with a particular focus on the first three months of life. To extend the growth trajectory analysis, the study includes several available specimens at older ages, collected at approximately 12 months for Yucatans and eight months for standard pigs. The specific aims were (i) to quantify the overall skull size and shape changes in Yucatan and standard pigs, through a series of linear, angular and volumetric measurements; (ii) to examine the ontogenetic growth trajectories that lead to craniofacial morphological variations between breeds through three-dimensional geometric morphometric analyses and (iii) to assess calvarial thickness changes and cranial joint maturation, providing insights into the role of joint development in shaping craniofacial morphology in both breeds.

## 2. Material and methods

### (a) Data collection and image processing

**Figure 1.**
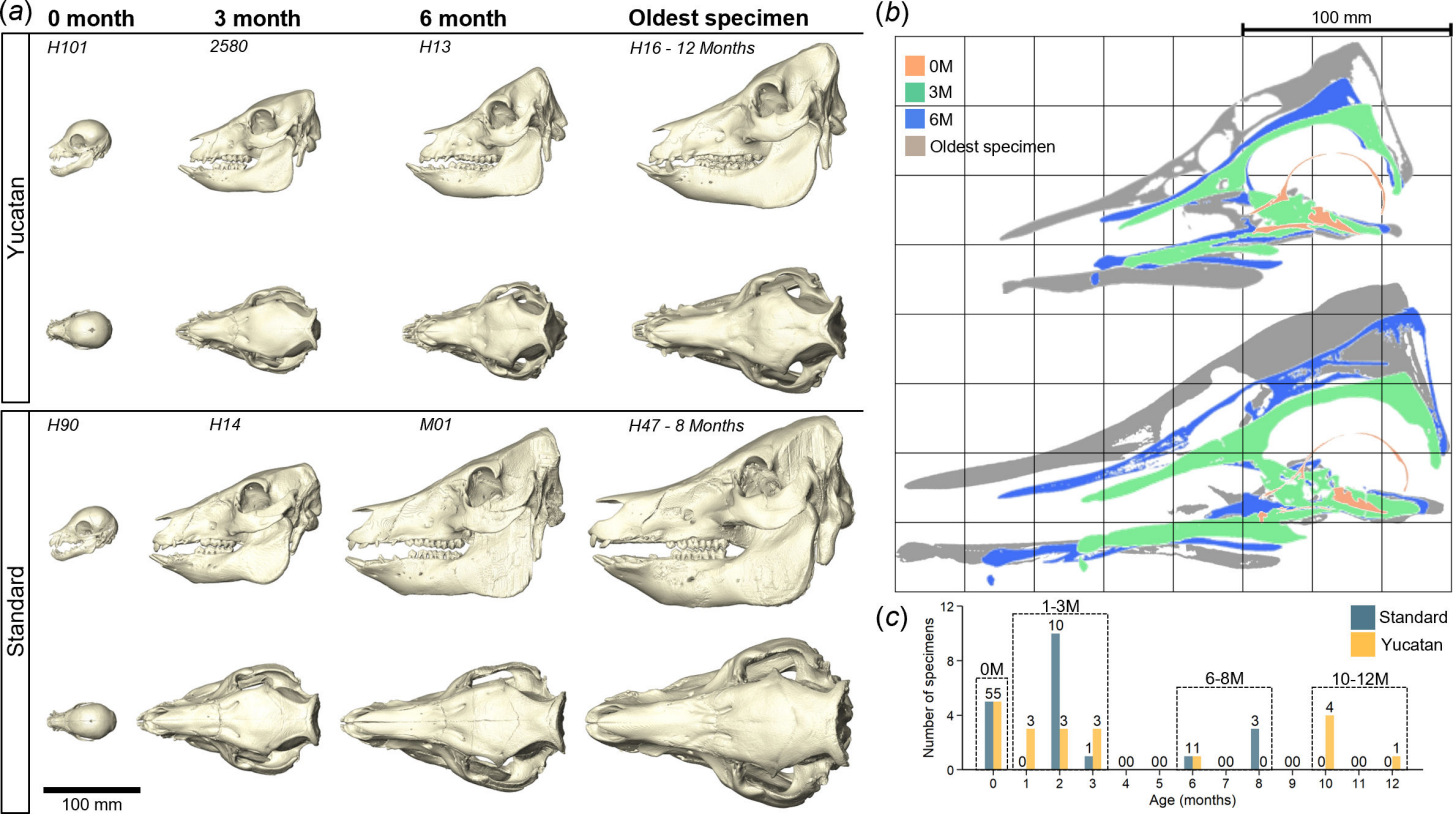
Overview of the head CT datasets of Yucatan miniature and standard pigs. (*a*) *In vivo* reconstructed skulls at specific ages. (*b*) Cross-sections of pre-aligned *in vivo* skulls in mid-sagittal plane. (*c*) Distribution of a total of 40 head specimens from birth to 12-month-age within four defined age groups used in the following analyses. See electronic supplementary material, table S1 for more details of the *in vivo* dataset.

Forty available intact head specimens of Yucatan pigs (*n* = 20, age: 0−12 months, whole-body weight: 0.4−57.5 kg) and standard (domestic) pigs (*n* = 20, age: 0−8 months, whole-body weight: 0.5−160.1 kg), without noticeable defects, were used in this study (figure 1; see electronic supplementary material, figure S1 for sex and age–weight relations). Yucatan miniature pigs were obtained from Premier BioSource (Ramona, CA), and standard pigs represent a mix of non-miniature breeds: 0−6 months old pigs were Yorkshire/Landrace mix breed obtained from either Premier BioSource (Ramona, CA) or Progressive Swine Farms (Woodinville, WA), and eight-month-old pigs were purebred Durocs breed obtained from Stein and Stewart Genetics (Odessa, MO) [14]. These specimens were collected between 2019 and 2023 for clinical training and experimental purposes and digitized using micro-computed tomography (CT) scanning, NSI X5000 at University of Washington and Nikon XT H 225 at University College London (see electronic supplementary material, table S1 for breed details and CT resolutions of each specimen). Three-dimensional models (with separated cranium and mandible) were reconstructed from CT images and oriented in same position (electronic supplementary material, figure S2), using Avizo software (v. 2022.1, Thermo Fisher Scientific, MA, USA) for craniometric measurements and morphological analyses.

### (b) Landmarking

**Figure 2.**
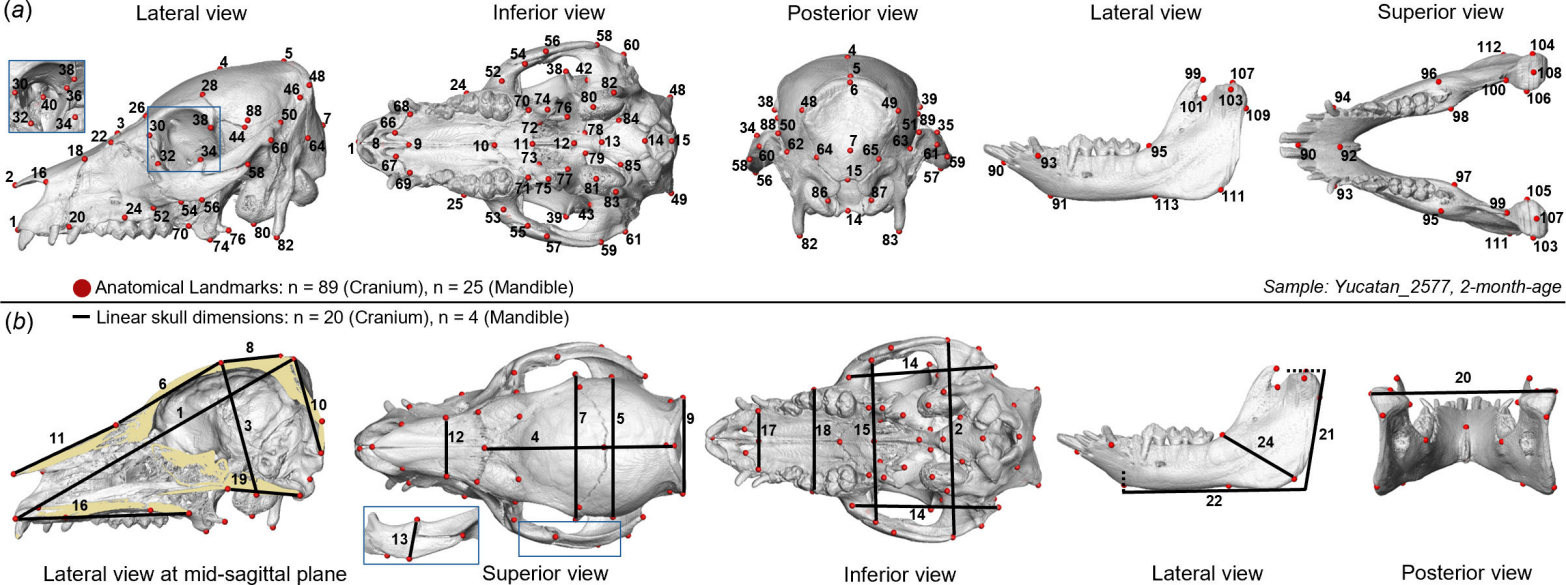
Illustration of anatomical landmarks (LMs) and linear measurements. (*a*) Eighty-nine LMs placed on the cranium and 25 LMs on the mandible (in red). (*b*) Twenty-four linear measurements derived from LMs. See electronic supplementary material, tables S2 and S3 for definitions of LMs and linear measurements. Zoomed-in views (blue box) highlight the LM position or measurement at specific regions.

A total of 114 anatomical landmarks (LMs; 89 on cranium and 25 on mandible) [17,18,20,22–24] were placed on each specimen by locating the distinct features of skull bones and joints (figure 2 and electronic supplementary material, table S2 for LM definitions). LM placement was conducted by a single investigator, repeating three times for each specimen to reduce bias, using Avizo software.

To perform a detailed characterization of skull morphology, surface semi-landmarks (SLMs), lying on the external surface, were created separately on the cranium and mandible of each specimen according to a prior semiautomated protocol [20] using the R packages *Arothron* [25] and *Morpho* [26]. First, the external surface meshes of the restructured cranial and mandibular models were extracted using the R function *out.inn.mesh* [27] and modified using Geomagic Wrap (v. 2017, 3D Systems Inc., NC) for smoothing and enclosing the extracted meshes [20]. Then, based on the Procrustes distances calculated using LMs, specimen no. 2577 (Yucatan, two-months-old) was selected as the template to seed semi-landmarking, as its cranium and mandible were most similar to the mean shape. SLMs were initialized on the cranial and mandibular surfaces of this template separately, resulting in 454 SLMs across the regions of face, cranial vault, palate and squamous and basilar parts of occipital bone, and 220 SLMs across the external and internal surfaces of the ramus with the mandibular body. Then, template sets were applied separately onto the cranial and mandibular surfaces of each specimen with SLM sliding permitted during distribution, generating a full configuration of 788 LMs per specimen (see electronic supplementary material, figure S3).

### (c) Craniometric measurements

Skull size and shape changes were assessed through a series of two-dimensional and three-dimensional measurements. These include a list of 24 standardized linear measurements [16,17,20,24] capturing the size changes of entire cranium and specific regions of face, cranial vault, skull base and mandible, mostly computed using LM coordinates (figure 2). Three skull indices, cranial module (CM) [20], cephalic index (CI) and facial–palatal index (FPI), derived from linear dimensions, serve as simple estimators of overall cranial size, neurocranial shape and facial shape, respectively. To evaluate the flexion of calvarium and skull base, two new angular measurements were carried out in this study, named fronto-parietal angle (*A*_B_) and spheno-occipital angle (*A*_SOS_). *A*_B_ is defined as the inclination between frontal and parietal bones, derived from *Nasion*, *Bregma* and *Lambda* points, and *A*_SOS_ captures the rotation of skull base at spheno-occipital synchondrosis (SOS), derived from *Hormion*, *Sphenobasion* and *Basion* points. The third classic mandibular angle (*A*_Go_) [24] is formed at the intersection between inferior border of the mandibular body and posterior border of the ramus. To characterize the size changes of key craniofacial anatomical cavities, intracranial volume (ICV), bilateral orbital volumes (OVs) and nasal cavity volume (NCV) were measured using R-based tools, *Icex* [28] and *endomaker* [29].

### (d) Morphometric analyses of skull form and shape

Morphological variations in the form (size and shape) [30] and shape alone of cranium and mandible were investigated using the R packages *Arothron* [25] and *Morpho* [26], following standardized procedures [20]. First, the reconstructed cranial and mandibular models from all specimens were subjected separately to Procrustes superimposition through generalized Procrustes analysis (GPA). GPA of the cranium utilized the dataset of 543 LMs per specimen whereas GPA of the mandible used the dataset of 245 LMs, both resulting in the matrices of shape variables (comprizing the coordinates of analysed LMs after size standardization) and centroid sizes (a geometric scale representing the original size of each specimen) [30].

To explore the modes of surface variations in the cranium and mandible during ontogeny, principal component analysis (PCA) was performed using the matrix of shape variables augmented with the natural logarithm of centroid sizes (form analysis) or using the matrix of shape variables only (shape analysis). This led to PCAs of cranial form, mandibular form, cranial shape and mandibular shape. To relate the variations representing by vectors of principal components (PCs) to morphology, the template surface meshes of the cranium and mandible (adopted from specimen no. 2577, two-month-old Yucatan) were warped along the vector to the extreme values of each PC of form or shape analysis. Then, the localized area differences between two warped surface meshes, representing form or shape at extreme PC scores, were computed and visualized through a colour map indicating regions of relative expansion and contraction along the vector of PC. PCs explaining less than 5% of total variances were ignored for visualization. Surface warping and colour mapping were all based on LMs and SLMs, and the apparent changes in the regions without any type of landmarks should be treated as approximations.

To evaluate the effect of applying dense SLMs on yielding outcome of shape and form analyses [31–33], an additional comparison was included in this study by performing PCA using either LMs only or the full configuration of LMs and SLMs, in both shape and form analyses of cranium and mandible, resulting in eight separated PCAs. To further quantify the differences between the two approaches, Pearson correlation analysis was applied to assess the association between the distance matrices [32,33] computed from the PC scores obtained using LMs-only approach and the full configuration.

Allometry (size-related changes in shape) and development (age-related changes in shape) [30] of cranium and mandible were compared between breeds through multivariate regression of the shape or form matrices on the natural logarithm of centroid size (ln(CS)) or age. The significance of the angle (computed as the dot product) between multivariate vectors was tested using a permutation test (1000 permutations) [20].

To further explore how the cranium covaries with the mandible during ontogeny, two-block partial least squares analyses (PLS) [34] were carried out between cranial form (Block 1) and mandibular form (Block 2), and cranial shape (Block 1) and mandibular shape (Block 2). These PLS analyses used the same landmark configurations as PCAs.

### (e) Thickness quantification of cranial bones and joints

The quantitative analysis of thickness changes in cranial vault was conducted across specific regions of interest (ROIs) of calvarial bones, utilizing mid-sagittal and coronal sectioning planes. In the mid-sagittal plane, the ROI extended from the anterior frontal lobe, passing through *Bregma*, and reaching the posterior occipital lobe. In the coronal plane, the ROI spanned from *Bregma* to bilateral *Euryons*. To eliminate the effect of the complex bone–suture interface on thickness quantification, the smoothed external surface meshes of the cranium (previously used for semi-landmarking) and extracted ICV (previously used for volume measurement) were selected to represent the external and internal bony surfaces of the cranial vault. Surface outlines were initially generated from each sectioning plane, and the curves delineating the boundaries of each ROI were extracted from outlines using anatomical features. Then, each curve was discretized into 50 equidistant points using R function *equidistantCurve* [26], and the bone thickness was computed as Euclidean distance between pairs of points on the inner and outer borders (see electronic supplementary material, figure S4).

The size (gap width) of seven cranial joints across the calvaria and skull base regions was measured. These included interfrontal, sagittal, nasofrontal, coronal and lambdoid sutures, as well as the inter-sphenoidal and spheno-occipital synchondroses (ISS and SOS). The measurement was carried out using CT slices at 30 identified locations per specimen, and these locations were chosen at the proximal and distal ends and the middle of each joint on both sides [35], with reference to anatomical landmarks or the mid-sagittal plane (see electronic supplementary material, figure S5). The gap width of each joint was measured along its region of patency, at either anterior–middle–posterior or superior–middle–inferior sublocations according to the orientation of joint presentation in each CT slice. This resulted in three measurements per location and a total of 90 measurements for all seven joints of each specimen. To minimize interobserver bias, all thickness measurements were performed by a single investigator using Avizo software and independently verified by a second investigator.

### (f) Statistical analysis

Mann–Whitney U-test was used to compare the group differences in terms of angular dimensions, and calvarial and joint thicknesses, with a *p*‐value ≤ 0.05 considered significant. All analyses were conducted using R.

## 3. Results

### (a) Overall skull size, shape and craniofacial cavities

**Figure 3.**
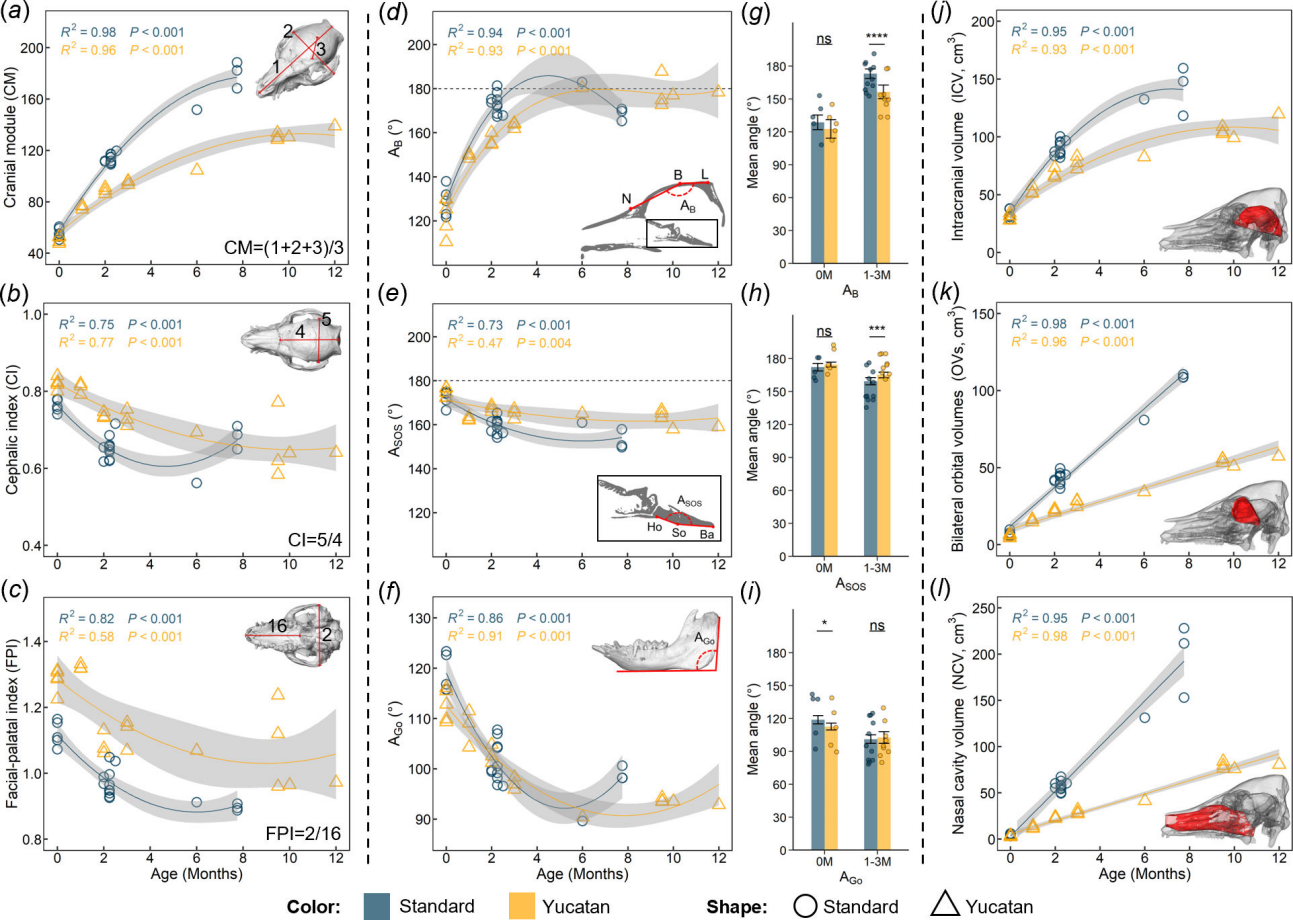
Summary of linear, angular and volumetric measurements of Yucatan miniature and standard pig skulls throughout the studied ages. (*a–c*) Changes in three key skull indices derived from linear measurements (shown in the top-right corner of each index figure). (*d–f*) Changes in the fronto-parietal angle (*A*_B_, derived from *Nasion*-N, *Bregma*-B and *Lambda*-L in mid-sagittal plane), spheno-occipital angle (*A*_SOS_, derived from *Hormion*-Ho, *Sphenobasion*-So and *Basion*-Ba in the mid-sagittal plane) and mandibular angle (*A*_Go_, derived from the inferior border of the mandibular body and posterior border of the ramus, measured at both sides with averages presented). (*g–i*) Quantification of differences in each angular dimension between standard and Yucatan pigs at birth (zero-month-old) and at age from one to three months. (*j–l*) Changes in intracranial volume (ICV), bilateral orbital volume (OVs) and nasal cavity volume (NCV) with age. See electronic supplementary material, figure S6 for the proportional contributions of each volume during ontogeny and volume–weight relations. Regression curves were derived from either linear or polynomial models and are reported with 95% confidence intervals (in grey). Significance symbols: ****p ≤ 0.0001, ***p ≤ 0.001, **p ≤ 0.01, *p ≤ 0.05, ns *p* > 0.05.

Figure 3 and electronic supplementary material, table S3 summarize the linear, angular and volumetric measurements for all specimens. Overall skull size (assessed by CM; figure 3*a*) of Yucatan and standard pigs is identical at birth, then the standard pig skull increases to approximately 1.36 and 1.41 times bigger than Yucatan pig skull at three and six months of age, following a nonlinear trend. For the size change in specific regions of cranial vault, base, face and mandible, the measured results indicate high similarity between both breeds at birth and the difference becomes pronounced after one month of age. For example, the mean difference in all 24 measured skull dimensions between both breeds is around 9% at birth and surges to 29 and 54% at age groups of 1−3 months and 6−8 months, respectively (see electronic supplementary material, table S3). Overall cranial shape (assessed by CI; figure 3*b*) becomes less rounded. The ratio of cranial breadth to length in Yucatan pigs decreases almost linearly from around 0.80 to 0.65 throughout the first 12 months of age, while that for standard pig shows a nonlinear association with age, decreasing from 0.80 at birth to 0.60 at four months then increasing to 0.65 at eight months, roughly. Overall facial shape was assessed by FPI (figure 3*c*). The ratio of skull breadth to palatal length (FPI) decreases from around 1.30 to 1.10 in Yucatan and from around 1.15 to 0.90 in standard pigs throughout the studied ages.

For angular changes, fronto-parietal angle (*A*_B_) is around 120° at birth in both breeds. Yucatan A_B_ stabilizes at 180° after six months of age, while *A*_B_ in the standard cranium increases to same level within the first three months and then decreases after six months (figure 3*d*). Spheno-occipital angle (*A*_SOS_) decreases by approximately 10° and 20° from 175° in the first six months of age in Yucatan and standard crania (figure 3*e*). Mandibular angle (*A*_Go_) varies similarly in both breeds in the first six months of age, decreasing from a maximum 125° to a minimum of 90° (figure 3*f*). As shown in figure 3*g*,*h*, mean *A*_B_ and *A*_SOS_ show no significant difference between breeds at birth, but statistical significance appears at 1−3 months. However, the comparison of mean *A*_Go_ between breeds shows the opposite result with a weak significant signal only found at birth (figure 3*i*).

For volumetric changes, ICV follows a similar pattern to CM (figure 3*j*). Yucatan and standard ICVs are identical at birth and then increase rapidly up to six months of age, with a higher growing rate observed in standard ICV than Yucatan. Bilateral OVs and NCV increase linearly with age at different rates between breeds throughout the studied age range (figure 3*k*,*l*). The proportional contributions (expressed as a percentage of each volume to the sum of three volumes per breed per age) change over time. The proportion of ICV decreases while those of OVs and NCV increase with age in both breeds (electronic supplementary material, figure S6*a–c*). Our results also suggest that the volumetric changes in craniofacial cavities (ICV, OVs and NCV) show stronger associations with whole-body weight than with age with very similar rates in both breeds (electronic supplementary material, figure S6*d–f*).

### (b) Growth trajectories and morphological variations in skull form and shape

**Figure 4.**
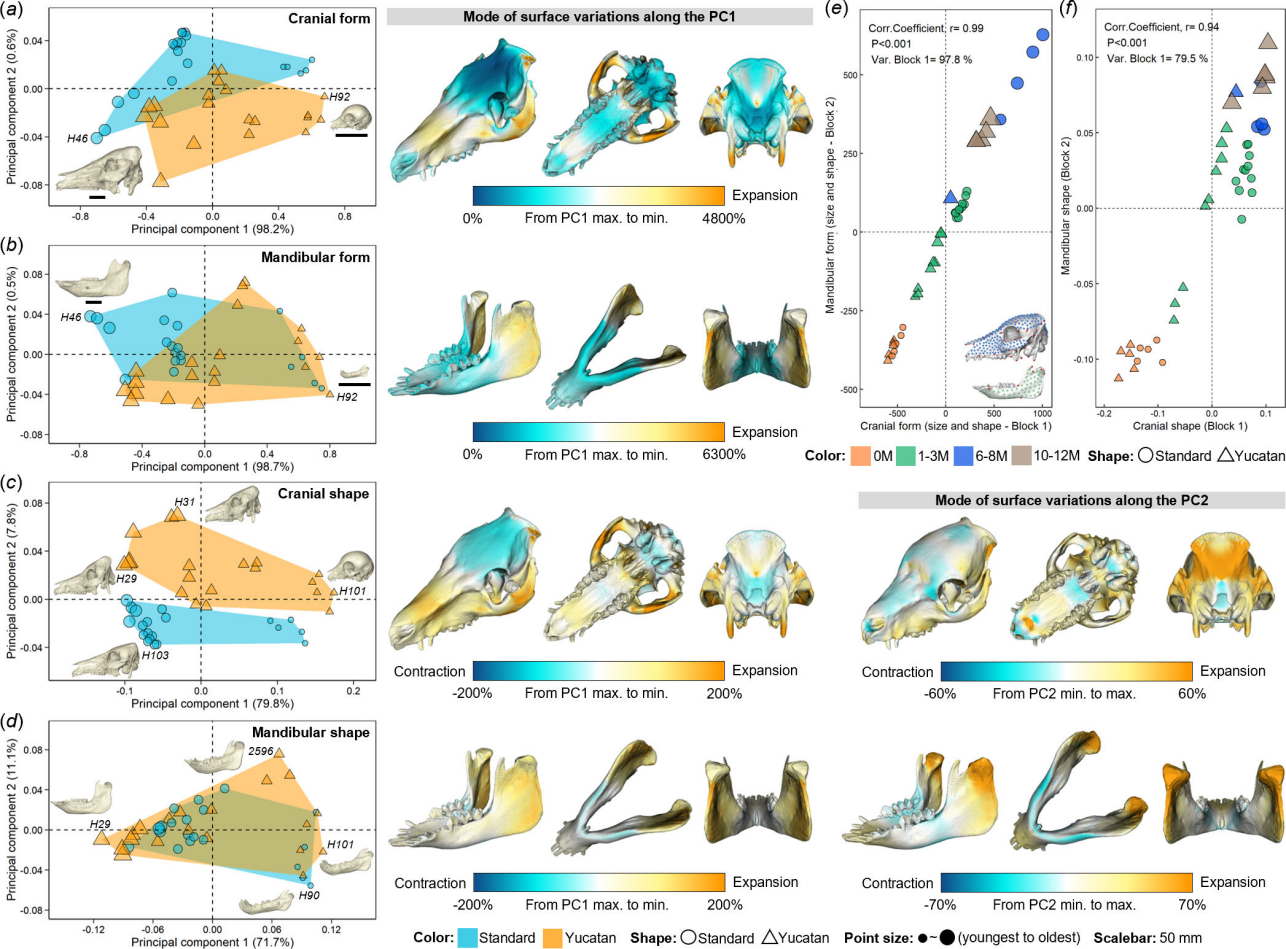
Morphological variations and ontogenetic growth trajectories. Principal component analysis (PCA) of (*a*) cranial form (size and shape), (*b*) mandibular form, (*c*) cranial shape and (*d*) mandibular shape, plotted with the first two principal components (PCs 1 and 2). The size-preserving models of cranium and mandible (in ivory) reconstructed from specimens representing the extreme PC1 scores are shown within the form PCA plots (*a*,*b*), and the coloured surface meshes (on the left) represent the form variations along PC1, by comparing the percentage local area differences of two surfaces warped at the extreme values of PC1. For shape PCA plots (*c,d*), the model of cranium or mandible (shape-preserving) and coloured surface meshes are presented for PCs 1 and 2, considering the variance explained by both PCs. Partial least squares (PLS) analyses between (*e*) cranial form (Block 1) and mandibular form (Block 2), and (*f*) cranial shape (Block 1) and mandibular shape (Block 2). All PCA and PLS were performed using the landmark configurations on cranium (89 LMs in red; 454 SLMs in light blue) and/or on mandible (25 LMs in red; 220 SLMs in light green) of all specimens. See electronic supplementary material, figure S3 for landmark configurations, S8 for comparison of performing PCA with or without using SLMs and S9 for breed-separated PLS.

PCA and PLS analyses were carried out to characterize the variations in form and shape of the cranium and mandible during postnatal ontogeny (figure 4), using the full landmark configurations of all Yucatan and standard specimens (electronic supplementary material, figure S3).

Notably, the correlation analysis shows highly significant associations between distance matrices derived from LMs-only approach and full configuration (*p*‐value < 0.0001, *r* > 0.98 for all shape and form analyses; electronic supplementary material, figure S7), which indicates the shape or form variation patterns revealed by two approaches are similar. By comparing the PCA results derived from these two configurations, we found minimal differences in overall distribution patterns of both breeds according to PCs 1 and 2 scores and the total variance explained by major PCs (>5%) in form and shape analyses of cranium or mandible, while the inclusion of SLMs captured more cranial shape variations (PC1 = 70.6% using LMs only versus PC1 = 79.8% using full configuration; electronic supplementary material, figure S8). This enables more accurate visualization of shape differences [33].

For both cranial and mandibular form PCAs, the first PC accounted for over 98% of the total variance with a far smaller proportion presented by PC2 (approx. 0.5%), and scores on PC1 strongly reflect how the allometric scaling of cranium and mandible differs between breeds (figure 4*a*,*b*, first column). The surface warping of the mean to extremes of PC1 indicates that the form differences from maximum to minimum PC1 scores (youngest to oldest) are mainly associated with overall size increase, with relatively large expansion across the nasal, maxillary, zygomatic, anterior palatal and posterior occipital regions of the cranium. For the mandible, the size of the ramus increases relative to the size of the body of mandible (figure 4*a,b*, second column).

For the PCA of cranial shape, the first two PCs explained for almost 90% of total variance (PC1 = 79.8% and PC2 = 7.8%), and scores on both PCs indicate that the cranial shapes of Yucatan and standard pigs vary in a similar allometric mode along PC1, whereas there are distinct differences between breeds along PC2 (figure 4*c*, first column). Positive PC1 scores are associated with a relatively long face and flattened cranial vault, with the opposite for negative scores. Cranial shape changes along PC2 from negative to positive values are mainly characterized by a relative contraction of the upper calvarium and expansion of posterior occipital regions (figure 4*c*, second and third columns). Variations in mandibular shape between breeds are extremely consistent with PCs 1 and 2 explaining 71.7 and 11.1% of total variance, respectively (figure 4*d*, first column). From positive to negative values of PC1, the entire ramus is expanded, while the original shape in other regions is preserved except a slight contraction found across the base of the mandibular body (figure 4*d*, second column). The shape variation assessed by PC2 reflects the expansion of the condylar process accompanied by contraction across the middle of the mandibular body (figure 4*d*, third column-).

The comparisons between breeds of ontogenetic trajectories from multivariate regressions are summarized in electronic supplementary material, table S4. The regressions of cranial shape on ln(CS) (allometry) are significant, explaining 76% of total variance in standard pigs and 83% in Yucatans, with trajectories significantly diverging between breeds (angle = 15.4°, *p*‐value = 0.024). The regressions of mandibular shape on ln(CS) show approximately 70% variance explained within each breed and also differ significantly. Cranial shape regression on age (development) explain >60% variance in Yucatans but only around 45% in standard pigs, with no breed differences in ontogenetic trajectory (angle = 19.8°, *p*‐value = 0.113). For mandible, age-related regressions explain similar shape variance as these on cranium, but trajectories significantly diverge. Moreover, the regressions of cranial or mandibular form on ln(CS) and age are all significant, explaining far greater proportions (approx. 99%, size; approx. 75–81%, age) of total variance in each breed, showing significant trajectory differences between breeds (4.0–6.2°, angle; *p*-values < 0.05).

The two-block PLS analysis between cranial and mandibular forms exhibits a significant correlation between the first PLS axis of each block (*r* = 0.99, *p*‐value < 0.001) and accounts for 97.6% of the total covariance between blocks (figure 4*e*). When limited to shape, although the correlation coefficient of cranial and mandibular shapes is 0.94 (*p*‐value < 0.001), the covariance between blocks is around 80%, and PLS scores indicate that the Yucatan and standard pigs share almost same level of covariance between cranial and mandibular shapes at birth (figure 4*f*). Through the breed-separated PLS, Yucatan and standard pigs exhibit a similar pattern of covariation (electronic supplementary material, figure S9).

### (c) Changes in the calvarial bone thickness

**Figure 5.**
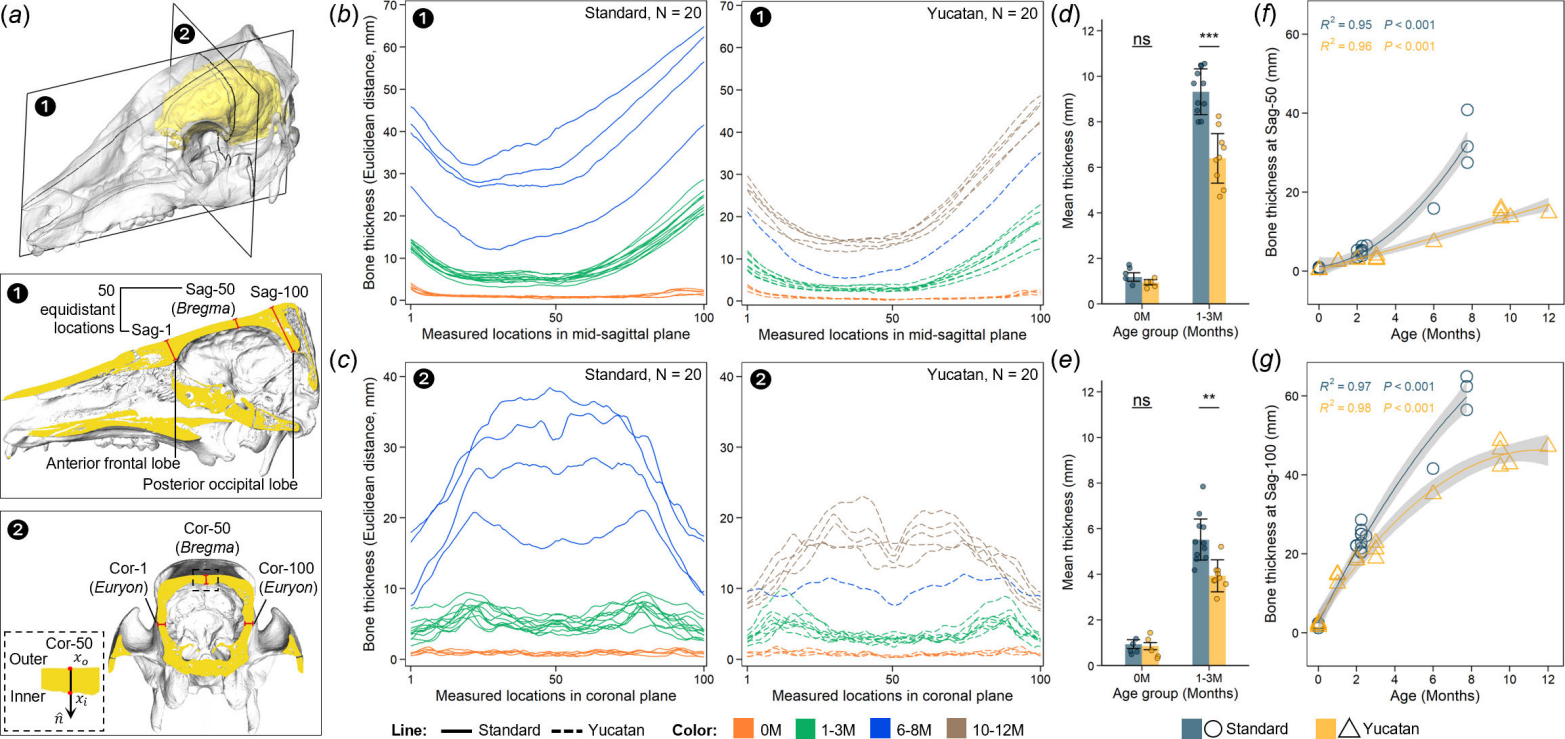
Changes in calvarial thickness during postnatal ontogeny. (*a*) Schematic diagram showing the regions of interest (ROIs). The ROI for the mid-sagittal plane (Sag) spans from anterior frontal lobe (Sag−1), passes through *Bregma* (Sag−50) and ends at posterior occipital lobe (Sag−100). The ROI for the coronal plane (Cor) extends from *Bregma* (Cor−50) to bilateral *Euryon* (Cor−1 and Cor−100). Points located on the outer and inner borders ($x_{o}$ and $x_{i}$, in red) highlight the boundaries of each ROI. Bone thickness was computed as Euclidean distance between outer and inner borders of each ROI, and 50 equidistant measures were taken between each two anatomical locations in the mid-sagittal plane (*b*) and coronal plane (*c*). Quantification of differences in calvarial thickness measured at mid-sagittal plane (*d*) and coronal plane (*e*) between standard and Yucatan pigs at birth (zero-month-old) and at age from one to three months. Thickness changes with age at specific locations of Sag−50 (*f*) and Sag−100 (*g*). Regression curves in (*f*,*g*) were derived from either linear or polynomial models and are reported with 95% confidence intervals (in grey).

The overall thickness distribution of the cranial vault across the predefined ROIs in the mid-sagittal and coronal planes and the thickness changes at specific locations are presented in figure 5. Our results show that calvarial thickness distribution is relatively uniform in both breeds at birth (depicted in orange in figure 5*b*,*c*), and the mean thickness values are similar within the range of 0.9−1.2 mm in both planes (figure 5*d*,*e*).

During the first three months of life, the cranial vault of both breeds exhibits notable alterations in overall morphology, transitioning into a phase of rapid and heterogeneous increase in thickness. The differences in calvarial thickness become significant between breeds, with mean values of around 5.5 and 9.3 mm in the mid-sagittal plane and 6.4 and 3.9 mm in the coronal plane for Yucatan and standard pigs, respectively, at ages of 1−3 months (figure 5*d*,*e*). In particular, from a sagittal view, calvarial thickness increases the most in the posterior cranial vault (above occipital lobe), anterior frontal region (above the frontal lobe) and then the middle of the cranial vault (around *Bregma*); from a coronal view, the cranial vault is thickened from the middle (around *Bregma*) to the area of maximum curvature of the parietal bone, with lesser change on the sides (around *Euryon*). The overall distribution remained relatively symmetrical (depicted in green in figure 5*b*,*c*). Subsequently, the changes in calvarial thickness of these two breeds continue to follow this pattern, albeit at different rates (depicted in blue and brown in figure 5*b*,*c*; electronic supplementary figure S10*a,b*). For example, the thickness at *Bregma* of the Yucatan cranium increases steadily from 4.4 to 16.9 mm (3–12 months), whereas that of the standard cranium increases at a higher rate, reaching 30.1 mm by eight months (figure 5*f*). Thickness at the posterior occipital lobe increases nonlinearly, from 22.9 to 46.3 mm (3–12 months) in Yucatans and from 29.1 to 66.0 mm (3–8 months) in standard pigs (figure 5*g*). Our results also indicate that calvarial thickness of Yucatan and standard pigs varies in a more consistent pattern with ICV than age (electronic supplementary material, figure S10*c–f*).

### (d) Morphological characterization of cranial joints

**Figure 6.**
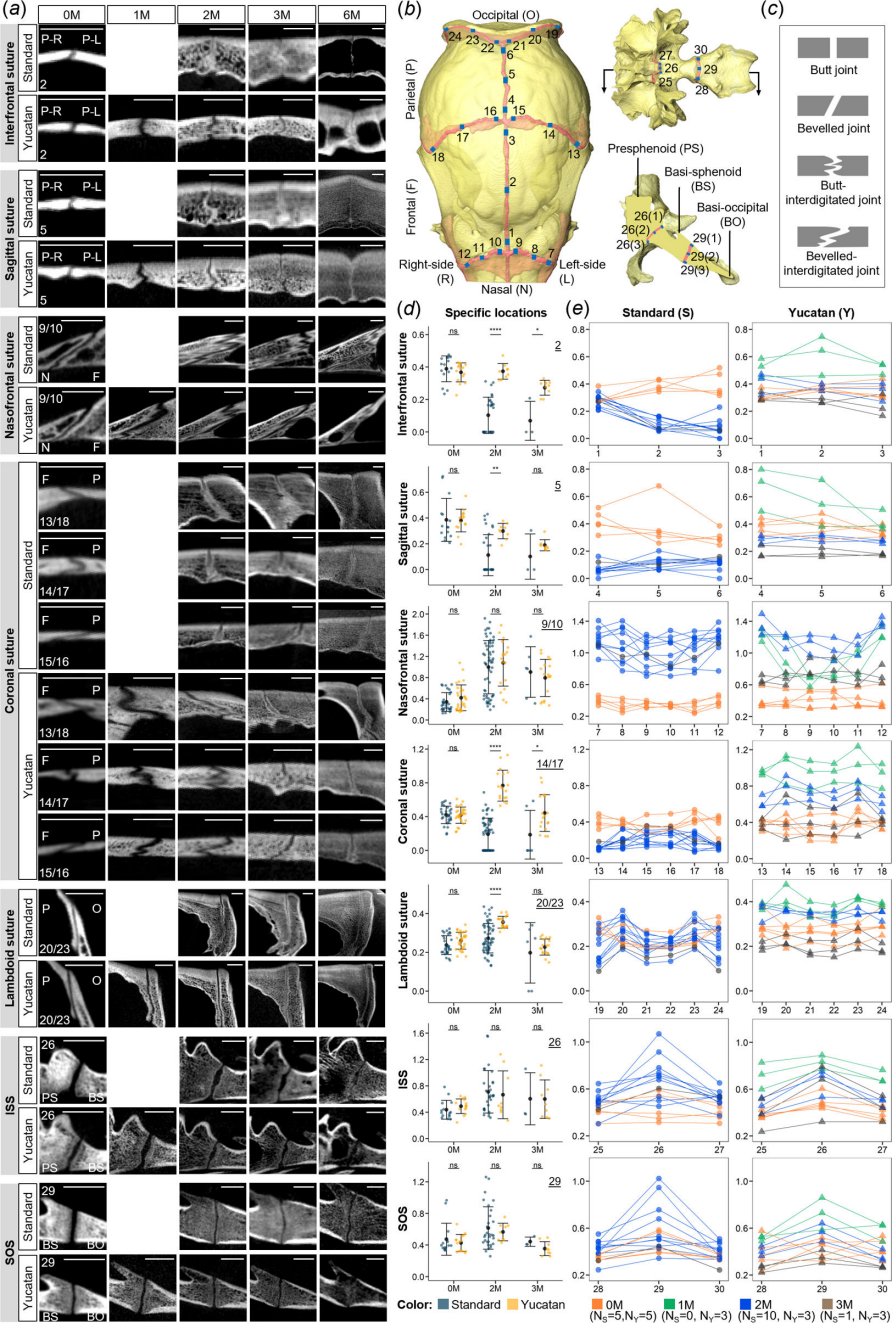
Morphological variations and gap width quantification of cranial joints. (*a*) CT images showing the changes in overall morphology of seven cranial joints at selected locations from zero to six months of age. All images were taken from the left side of the skull. Scale bar, 5 mm. (*b*) Thirty defined locations of CT slices (in blue) for gap width measurements. Measurements were taken at three sublocations along patent region of each joint seen on each CT slice. See electronic supplementary material, figure S5 for definitions of each location. (*c*) Four typical types of joint morphology. (*d*) Comparison of gap width at selected locations (underlined number on top-right corner) between standard and Yucatan pigs. Each point represents a single measurement (three for each location and 90 for each specimen). Error bars represent standard error of the mean. (*e*) Average gap width changes along the joint over the first three months of age. Each point represents the average widths of three measurements at one location and each line refers to one specimen. Significance symbols: ****p ≤ 0.0001, ***p ≤ 0.001, **p ≤ 0.01, *p ≤ 0.05 and ns *p* > 0.05. See electronic supplementary material, figures S11 and S12 for CT images at other locations and comparison of average gap widths.

Joint morphology in different cranial regions presents high variability during early development but shows relative consistent morphological features between breeds, with interdigitated sutures identified in the calvarial regions (except the lambdoid suture) and straight synchondroses within the skull base (figure 6*a*). There are four major shapes of cranial joint identified in CT cross-sectional slices at pre-defined locations, including butt, bevelled, butt–interdigitated and bevelled–interdigitated joints (figure 6*b*,*c*). Interfrontal and sagittal sutures form slightly bevelled connections at birth, become interdigitated within the first three months of age and exhibit straight interfaces at the fully fused stage (i.e. six months of age; figure 6*a* and electronic supplementary material, figure S11*a* and S1,1*b*). The nasofrontal suture shows increasing complexity of interdigitation along the midline to lateral direction (i.e. from location 9 to 7) and with age until ossification is completed (figure 6*a* and electronic supplementary material, figure S11*c*). The coronal suture exhibits the most unique structure among all assessed joints, characterized as twisted morphology, where the frontal bone front initially lies above the parietal bone and their positions gradually reverse in intertwining connections from the midline to the lateral regions (i.e. from locations 15,16 to 13,18; figure 6*a*). The lambdoid suture and two skull base synchondroses (ISS and SOS) are simple modifications of butt or bevelled joint morphology. The ISS and SOS remain patent at lower closure rates until six months of age (figure 6*a* and electronic supplementary material, figure S11*d*).

The overall patency of different joints between the two breeds was quantified through the gap width measurements. Our results suggest that the calvarial sutures experience higher closure rates in standard pigs than those of Yucatan pigs after birth, based on significant differences (*p*-values ≤ 0.05) in average gap widths, while skull base synchondroses show no obvious difference (*p*-values > 0.05) (electronic supplementary material, figure S12). Notably, the skull base synchondroses show relatively uniform ossification at different sublocations, whereas the sutural joints, except for the lambdoid suture, exhibit high variability in gap width at different sublocations and non-uniform, region-specific closure (figure 6*d*,*e*). Specifically, the interfrontal, sagittal and coronal sutures in standard pigs are fused endocranially by two months of age, contrasting with the patent gaps observed in same-age Yucatan pigs (figure 6*a*,*d*,*e*).

## 4. Discussion

### (a) Changes in craniofacial cavities and overall cranial size

Postnatal growth of the cranium is associated with the development of craniofacial organs and cavities. In this study, the absolute and proportional changes in volumes available to house the brain, eyes and nasal cavity were assessed because of likely roles of these organs in driving morphological change, as observed in other growing mammalian crania [20,36]. Our study highlights the allometric growth of intracranial volume (ICV) and the relatively linear growing trends of bilateral orbital volumes (OVs) and nasal cavity volume (NCV) from birth to the age of sexual maturity in Yucatan and standard pigs. Both breeds demonstrate proportional decrease in ICV, proportional increase in NCV and a lesser proportional increase in OVs. These dynamics emphasize a pivotal role of brain growth in directing neurocranial growth and similarly a strong involvement of nasal cavity expansion in shaping the facial skeleton, while to a lesser extent, the contribution of eye socket volumes to the regional growth of face. Bigger neurocranial volumes often correspond to more extensive facial structures [37], suggesting the discrepancies in volumes of these craniofacial organs, and cavities may underlie the breed-specific size differences that emerge with age between Yucatan and standard pigs. We found that the development of these craniofacial cavities covaried more tightly with whole-body weight than with age and that the growth pattern of each assessed volume in the two breeds matched at equivalent weight (see electronic supplementary material, figure S6*d–f*).

### (b) Allometric growth trajectory and morphological diversification

Concerning ontogenetic allometry, our study suggests distinct phases of acceleration and deceleration in form (size and shape) changes of cranium and mandible of Yucatan and standard pigs during the first year of life. From zero to three months of age, both breeds exhibit greater changes in cranial and mandibular form compared with subsequent periods, reflecting an allometric growth trajectory. Form variations in these bones are dominated by size changes during the first year of life, and there is minimal divergence regarding the mode of variation between breeds. Typically, as overall size increases, facial dimensions (especially the length) scale positively, while the neurocranial dimensions scale with negative allometry, emphasizing the developmental interplay between the face and braincase in mammals [38–40]. When focusing on shape-related changes, we found that Yucatan and standard pigs exhibit nearly the same mandibular shape throughout the studied range. Standard pig crania tend to develop a slender face and flat head, while the Yucatan cranium is characterized by a more brachycephalic shape, featuring a shortened face and a relatively concave dorsal outline. In Yucatans, the shape of the midface varies mainly in response to the development of the nasal cavity and its associated tissues [14]. In contrast, neurocranial shape appears to be governed by factors beyond brain development, evidenced by the non-uniform calvarial thickening patterns observed in this study. The epigenetic action of masticatory and occipital muscles may explain some morphological changes in the areas of their attached sites [18,41]. For instance, the posterior cranial vault becomes more prominent over time along with wider zygomatic arches, and larger and more upright mandibular rami, which perhaps increases space for masticatory muscles as they adapt for increased functional demands of mastication [42]. Moreover, diet and environmental conditions also significantly influence the craniofacial development, as the morphological variations between domestic and wild crania initialize before birth and become more pronounced through distinct postnatal ontogenetic trajectories [18,19,42]. In this study, domestic phenotypes show nearly identical cranial forms at birth, with divergence emerging progressively during ontogeny, which may reflect early developmental alternations and offer insight into the mechanisms underlying domestication-related morphological changes [19]. Interestingly, recent findings suggest that specific developmental mechanisms (e.g. heterochrony) may obscure typical allometric patterns, particularly in response to extreme biomechanical or ecological pressures [43]. While our study focuses on intraspecific variation under domestication, similar developmental pattern may also contribute to craniofacial disparity in broader evolutionary contexts.

### (c) Joint morphology and maturation mode

We found that cranial sutures in calvarial regions are patent at birth, with similar sutural gaps observed between breeds. However, the calvarial sutures in standard pigs become gradually narrower compared with those in Yucatans, as indicated by the available measurements at two and three months of age. Although comparative data for one-month-old specimens were lacking, it is reasonable to infer that this trend was present in the first month of life. Interestingly, a prior study conducted by Rafferty *et al.* [13], who utilized microscopic imaging for sutural width quantification [44], reported contrasting findings in older pigs (4–5 months old), that the nasofrontal and coronal sutures in Yucatans had narrower gaps than same-age standard pigs. This finding may reflect altered timing of calvarial suture closure in Yucatans. In the younger pigs, we observed an increasing degree of interdigitation in the coronal suture of Yucatans, which may indicate delayed maturation relative to standard pigs (electronic supplementary material, figure S13), while the nasofrontal suture exhibited a coarser and less interdigitated structure, which may account for the growth constraint in the face.

The premature cessation of growth of the skull base is widely recognized in the syndromic forms of craniosynostosis [45], and early fusion of skull base synchondroses facilitates the progression of craniofacial dysmorphology in mouse models [21]. However, although the Yucatan pig has the phenotype of midfacial hypoplasia [13], open joints (inter-sphenoidal and spheno-occipital synchondroses—ISS and SOS) persist at the base, occurring throughout postnatal calvarial suture maturation, indicating that skull base elongation is not a direct driver of facial shortening in this breed. Theoretically, the prolonged patency of skull base joints may allow for the continued base expansion, mitigating early growth constraints on midface. Nevertheless, we observed the diminished growth of facial cavities and delayed fusion of calvarial sutures as well as the shape-preserved mandible in Yucatans compared with standard pigs. Thus, these findings highlight differential growth patterns of the midface and neurocranium during postnatal ontogeny. The distinct craniofacial characteristics of Yucatan pigs likely arise from a complex interplay of genetic, developmental and biomechanical factors [46] but not premature skull base fusion.

### (d) Limitations

This work has several limitations, and the key ones are: (i) although this study primarily examines postnatal craniofacial growth and development, there is still a lack of age-matched specimens between breeds within the first three months of age (e.g. no one-month-old standard pigs), and the specimens are not well distributed throughout the entire studied age range due to data availability. (ii) LM-based morphometric analyses were only carried out on the external surfaces of each cranium and mandible, and the surface warping/area differences of unlandmarked regions (e.g. teeth or anterior skull base) are less accurate [20]. (iii) Considering the quality of each CT dataset and structural complexity, the thickness and gap quantifications of craniofacial bones and joints did not include most facial and skull base regions, which will be addressed in our future work.

## 5. Conclusion

In summary, this study characterizes the skull growth trajectory and craniofacial morphological variations between Yucatan miniature and standard pigs during postnatal ontogeny. Our findings show that postnatal skull growth in both breeds is allometric and dominated by increasing size of craniofacial organs and capsules. The overall skull form (size and shape) of both breeds is similar at birth, but their skull shapes diverge during ontogeny, with Yucatans developing a relatively short face and compact neurocranium while maintaining similar mandibular shape to standard pigs. Over the first three months of life, calvarial sutures of Yucatans present delayed fusion compared with standard pigs, while the skull base synchondroses (ISS and SOS) remain patent in both breeds. Together, these findings underline the differential growth patterns of midface, neurocranium and mandible, and their associations with organ development, cavity expansion and joint maturation, thus offering insights into the mechanisms driving craniofacial diversification in pig models.

## Data Availability

All data generated or analysed during this study are included in the article and electronic supplementary material [47]. All raw data, including cranial measurements and morphometric datasets, along with a fully annotated R script, are stored in the Dryad Digital Repository [48], supporting the reproduction of statistical analyses presented in this study. Supplementary material is available online [47].
